# Risk Profiles for Barrett’s Esophagus Differ between New and Prevalent, and Long- and Short-Segment Cases

**DOI:** 10.1371/journal.pone.0169250

**Published:** 2016-12-30

**Authors:** Mimi C. Tan, Jackson Murrey-Ittmann, Theresa Nguyen, Gyanprakash A. Ketwaroo, Hashem B. El-Serag, Aaron P. Thrift

**Affiliations:** 1 Section of Gastroenterology and Hepatology, Department of Medicine, Baylor College of Medicine and Michael E. DeBakey Veterans Affairs Medical Center, Houston, Texas, United States of America; 2 Center for Innovations in Quality, Effectiveness, and Safety, Michael E. DeBakey Veterans Affairs Medical Center, Houston, Texas, United States of America; 3 Dan L Duncan Comprehensive Cancer Center, Baylor College of Medicine, Houston, Texas, United States of America; Shiga University of Medical science, JAPAN

## Abstract

**Background:**

Previous studies on Barrett’s esophagus (BE) risk factors have had differing case definitions and control groups. The purpose of this study was to examine differences in risk factors between newly diagnosed vs. prevalent BE, long- vs. short-segment BE, and endoscopy-only BE without specialized intestinal metaplasia (SIM).

**Methods:**

We conducted a cross-sectional study among eligible patients scheduled for elective esophagogastroduodenoscopy (EGD) and patients eligible for screening colonoscopy, recruited from primary care clinics at a Veterans Affairs center. All participants completed a survey on demographics, gastroesophageal reflux disease (GERD) symptoms and medication use prior to undergoing study EGD. We compared BE cases separately to two control groups: 503 primary care controls and 1353 endoscopy controls. Associations between risk factors and differing BE case definitions were evaluated with multivariate logistic regression models.

**Results:**

For comparisons with primary care controls, early onset frequent GERD symptoms were more strongly associated with risk of long-segment BE (OR 19.9; 95% CI 7.96–49.7) than short-segment BE (OR 8.54; 95% CI 3.85–18.9). Likewise, the inverse association with *H*. *pylori* infection was stronger for long-segment BE (OR, 0.45; 95% CI, 0.26–0.79) than short-segment BE (OR, 0.71; 95% CI, 0.48–1.05). GERD symptoms and *H*. *pylori* infection was also more strongly associated with prevalent BE than newly diagnosed BE. Few differences were observed between BE cases and endoscopy controls. Endoscopy-only BE was associated with GERD symptoms (OR 2.25, 95% CI 1.32–3.85) and PPI/H2RA use (OR 4.44; 95% CI 2.61–7.54) but to a smaller degree than BE with SIM.

**Conclusion:**

We found differences in the strength and profiles of risk factors for BE. The findings support that epidemiological studies of BE should make a distinction between long and short, new and prevalent, endoscopy-only and BE with SIM as well as type of controls.

## Introduction

Barrett’s esophagus (BE) is the only precursor for esophageal adenocarcinoma, a rapidly increasing and highly fatal cancer [1,2]. BE develops in 5–15% of individuals with symptoms of gastroesophageal reflux disease (GERD), and may affect 2% of the general adult population [3]. Assessment of BE risk factors in studies conducted over the past two decades have enabled better understanding of disease pathophysiology, and prevention; however, additional research and validation of BE risk factors are warranted.

Studies examining risk factors for BE have identified early onset of frequent GERD symptoms [4] and obesity [5,6] (in particular, visceral abdominal obesity [7]) as the strongest risk factors for BE. White race, male sex and older age have also been well described as risk factors for BE [8,9]. On the other hand, *Helicobacter pylori* infection is associated with lower risk for BE [10–12]. Emerging risk factors include lower gluteofemoral obesity [13] and shorter height [14]. However, studies of BE have reported conflicting results for associations with various other risk factors, including tobacco smoking [15,16], alcohol consumption [15,17], use of nonsteroidal anti-inflammatory drugs (NSAIDs) [18], bisphosphonates [19], as well as contrasting magnitudes of association with “known” risk factors [20].

Because the onset of BE is asymptomatic and diagnosis requires endoscopy and biopsy [21–24], there have been no cohort studies of incident BE, and such studies are unlikely to be forthcoming. Therefore, cross-sectional and case-control studies have exclusively been used to examine BE risk factors. Along with the choice of control group (e.g., population-based vs. clinical controls), the BE case definition may have influenced the results of these studies. For example, studies may have suffered from prevalence-incidence bias, and therefore missed important risk factors for BE by including predominantly or exclusively prevalent cases and systematically excluding newly diagnosed cases with concomitant neoplasia (dysplasia or cancer). Furthermore, long-segment BE confers worse outcomes, and may also reflect more or worse risk factors than short-segment BE. Finally, some studies have also included endoscopically visible BE irrespective of histological confirmation; however, presence of specialized intestinal metaplasia (SIM) may be distinct or more advanced in etiology from endoscopy-only BE. However, these hypotheses have not been extensively examined, and regardless of these shortcomings, current guidelines recommend screening for BE in those with chronic GERD and at least two risk factors, including age >50 years, white race, abdominal obesity, tobacco smoking history, and family history of esophageal adenocarcinoma [21,22]. A single study examining multiple case groups would be instructive for understanding BE risk factors.

We therefore present an analysis of risk factors for BE using data from a single, large cross-sectional study of BE patients in order to compare multiple BE case definitions (long- vs. short-segment; newly diagnosed vs. prevalent; SIM vs. endoscopy-only BE) with controls.

## Materials and Methods

### Study Population and Design

We conducted a cross-sectional study at the Michael E. DeBakey Veterans Affairs Medical Center (MEDVAMC) in Houston, TX from February 15, 2008 to August 20, 2013 to examine risk factors for BE [25]. In brief, we invited (1) consecutive eligible patients who were scheduled for an elective esophagastroduodenoscopy (EGD) at MEDVAMC to participate in the study; and (2) randomly selected patients eligible for screening colonoscopy from seven selected primary care clinics at the same hospital, who underwent the study EGD at the same time as their screening colonoscopy. All study participants had to be 40–80 years of age (and 50–80 years for primary care patients) and undergo a study upper endoscopy. The lower age limit in the primary care group was 50 as this is the age when screening colonoscopy is recommended to commence. The purpose of enrolling patients seen in primary care was to obtain controls without BE from the source population for BE cases at the Houston VA. These controls represent patients, who, if they had BE, would be diagnosed with BE at the Houston VA. None of primary care patients were primarily referred for EGD. The patients from endoscopy clinics are symptomatic and are typically undergoing an EGD to rule out BE. The same eligibility criteria were used for both groups. Patients with a previous history of gastroesophageal surgery, previous diagnosis of cancer (esophageal, lung, liver, colon, breast or stomach), currently taking anticoagulants, with significant liver disease (as indicated by platelet count < 70,000/mL, ascites, or known gastroesophageal varices), or a history of major stroke or mental disorder were ineligible for the study. Among eligible patients in the elective EGD group, 70% completed the study (underwent the study EGD and completed the study questionnaire). In the primary care group, 43% of eligible patients completed the study; however among patients who actually underwent their colonoscopy, 85% completed the study EGD and questionnaire.

This study was approved by the Institutional Review Board at Baylor College of Medicine (Board 4 for protocol H-21436) and the Office of Research and Development at MEDVAMC. Written informed consent was obtained from all participants prior to being interviewed for the study.

### Study Esophagogastroduodenoscopy

All study participants underwent the study EGD with systematic recording of suspected BE [26], hiatus hernia (absent, <3 cm, and ≥3 cm), and the Hill et al [27] classification of the gastroesophageal flap valve in the retroflexed endoscopic view (score range, 1–4). At least one targeted biopsy specimen was taken from suspected BE areas using jumbo biopsy forceps. BE length was determined by the Prague CM classification [26]. We performed gastric mapping by taking 7 mucosal biopsy samples from the antrum (from the greater and lesser curvatures), the corpus (from the distal greater, distal lesser, proximal greater, and proximal lesser curvatures), and the cardia [28].

### Case Definitions

For the current analysis, we performed additional review of the study histopathology reports and the electronic medical record for each study participant to define their BE cases status. For the overall analysis, we included 329 patients with BE. Where possible, we defined BE cases according to long- (≥3 cm) (n = 118) vs. short-segment (<3 cm) BE (n = 200), and newly diagnosed BE (first evidence of BE on study EGD) (n = 208) vs. prevalent BE (self-reported diagnosis of BE or history of BE diagnosis on review of the electronic medical record before the study EGD) (n = 109). A subject was considered to have definitive BE if SIM (confirmed by alcian-periodic acid-Schiff stain) under histopathologic examination was present in at least one biopsy sample obtained from tubular esophagus. Two expert pathologists reviewed all slides for suspected BE to determine the presence of SIM. Subjects with endoscopy-only BE (n = 85) were defined by the presence of suspected BE in the absence of SIM and were included in this analysis as a separate case group (“Endoscopy-only BE”).

### Control Definitions

We compared 329 BE cases separately with two control groups of patients without endoscopically suspected BE on their study EGD: (1) 503 patients recruited from primary care clinics (“Primary care controls”), representing the underlying source population from which cases arose; and (2) 1353 patients recruited from endoscopy clinics (“Endoscopy controls”), representing the population undergoing endoscopy from which cases are diagnosed.

### Data Collection

Study participants completed a computer-assisted survey before the study EGD. The survey ascertained information about social background, lifetime history and current use of alcohol and cigarette smoking, physical activity, medical history, onset, frequency and severity of GERD symptoms, and use of medications such as H2-receptor antagonists (H2RAs), proton pump inhibitors (PPIs), and non-steroidal anti-inflammatory drugs (NSAIDs). Race and ethnicity (non-Hispanic white, black, Hispanic, Asian, or other) were self-reported on the questionnaire and verified by manual review of the VA Computerized Patient Record System (CPRS). Height and weight were measured prior to the study EGD and were used to calculate body mass index (BMI; kg/m^2^). We calculated waist-to-hip ratio (WHR) and categorized participants into tertiles based on the distribution of WHR in the primary care controls.

*H*. *pylori* positivity was defined if organisms were seen on histopathology from any study gastric biopsy site, or if review of the medical record showed a previous positive biopsy, presence of serum antibodies, or treatment received. We examined and graded gastric biopsies for features of active and chronic gastritis and gastric atrophy according to the standardized operative link for gastritis assessment system [29], which uses the updated Sydney System [28]. A score of ≥1 on any biopsy from either the antrum or corpus was considered gastritis.

### Symptoms of Gastroesophageal Reflux Disease

We ascertained a history of GERD symptoms using a slightly modified version of the validated Gastroesophageal Reflux Questionnaire [30]. We asked participants about experience of heartburn (“a burning pain or discomfort behind the breastbone in your chest”) or acid regurgitation (“a bitter or sour-tasting fluid coming up into your throat or mouth”); positive responses to these initial screening questions elicited further questions about age at onset of symptoms and frequency of symptoms at ages 10–19, 20–29, 30–49, and 50–79 years, as applicable, on a five-point ordinal scale [4]. We defined participants as never having had GERD symptoms if they reported no symptoms of heartburn or acid regurgitation at all age periods; for all other participants, frequency and severity of GERD symptoms were equal to their highest reported frequency (and severity) of either heartburn or acid regurgitation. We defined “frequent symptoms” as those occurring at least weekly. Cumulative GERD symptom duration (years) was defined as the total number of years from age 10 to age at study recruitment in which a participant had frequent GERD symptoms, and was calculated by summing all age intervals where at least weekly GERD symptoms were reported.

### Statistical Analysis

Chi-square tests were used to examine differences between groups for categorical variables and t-tests were used for continuous variables. We calculated odds ratios (ORs) and 95% confidence intervals (CIs) for associations between an exposure variable of interest and the risk of BE using unconditional multivariate logistic regression models. All models were adjusted for age (40–50, 50-<60, 60-<70, 70–80 years), sex and race/ethnicity (white, black, other). Statistical significance was determined at *α* = 0.05, and all tests for statistical significance were two-sided. All analyses were conducted using SAS version 9.4 (SAS Institute, Cary, NC).

## Results

The mean age of the study population was 60.3 years (standard deviation, 8.1 y), and 91.9% were male. The majority were white (65.2%), 32.1% were black, and 2.7% were classified as other race/ethnicity. The mean BMI of the study population was 30.1 kg/m^2^ (standard deviation, 6.1 kg/m^2^). The demographic, anthropometric, and specific clinical characteristics of the BE cases (n = 329), primary care controls (n = 503), and endoscopy controls (n = 1353) are listed in S1 and S2 Tables. BE cases were significantly more likely to be white compared with either control group (both p<0.001). BE cases were more likely than primary care controls to have a history of GERD symptoms (p<0.001) and have a high WHR (p = 0.002), but less likely to have *H*. *pylori* infection (p<0.001) and gastritis (p<0.001) (S1 Table). BE cases were older (p<0.001) and significantly more likely to be male (p<0.001) than endoscopy controls (S2 Table).

Of the 318 BE cases with available data on Prague CM classification, 118 (37.1%) had long-segment BE and 200 (62.9%) had short-segment BE. Dysplasia was more frequent in cases with long-segment BE than short-segment BE (p<0.001). Among 118 with long-segment BE, 33.1% were found to have dysplasia on study EGD; while 11.1% of the 200 with short-segment BE had dysplasia. Among BE cases, we were able to classify 208 (65.6%) as being newly diagnosed with BE and 109 (34.4%) as prevalent BE cases. Among 208 newly diagnosed with BE on study EGD, 16.8% had concurrent dysplasia compared to 23.9% with dysplasia in those with prevalent BE (p = 0.30).

### Long-segment vs. Short-segment BE

Compared with primary care controls, patients with long-segment BE were significantly more likely to have early onset of frequent GERD symptoms (≥weekly symptoms of GERD before age 30 years vs. never GERD symptoms: OR, 19.9; 95% CI, 7.96–49.7) or used PPIs or H2RAs (OR, 6.90; 95% CI, 4.20–11.3), but less likely to have *H*. *pylori* infection (OR, 0.45; 95% CI, 0.26–0.79) or chronic gastritis (OR, 0.56; 95% CI, 0.38–0.88) (Table 1). Similar associations were seen for comparisons between primary care controls and short-segment BE cases (≥weekly GERD age <30 years: OR, 8.54; 95% CI, 3.85–18.9; *H*. *pylori* infection: OR, 0.71; 95% CI, 0.48–1.05; chronic gastritis: OR, 0.81; 95% CI, 0.56–1.15); however, the ORs for all risk factors were generally higher for long-segment BE than for short-segment BE.

**Table 1:** Associations with risk of Barrett’s esophagus, compared with primary care controls.

		All BEs (n = 329)	Long-segment BE (n = 118)	Short-segment BE (n = 200)	Newly diagnosed BE (n = 208)	Prevalent BE (n = 109)
		AOR* (95% CI)	AOR* (95% CI)	AOR* (95% CI)	AOR* (95% CI)	AOR* (95% CI)
BMI						
	<25	1.00 (Ref)	1.00 (Ref)	1.00 (Ref)	1.00 (Ref)	1.00 (Ref)
	25–29.9	0.75 (0.48–1.18)	0.50 (0.26–0.96)	0.92 (0.55–1.56)	0.61 (0.37–1.01)	1.18 (0.57–2.45)
	≥30	0.73 (0.47–1.13)	0.61 (0.33–1.12)	0.81 (0.49–1.36)	0.66 (0.41–1.06)	0.96 (0.47–1.96)
WHR						
	Tertile 1	1.00 (Ref)	1.00 (Ref)	1.00 (Ref)	1.00 (Ref)	1.00 (Ref)
	Tertile 2	0.99 (0.68–1.46)	1.01 (0.57–1.78)	0.93 (0.60–1.45)	0.90 (0.58–1.39)	1.16 (0.64–2.09)
	Tertile 3	1.19 (0.81–1.75)	1.22 (0.70–2.13)	1.14 (0.74–1.77)	1.04 (0.67–1.61)	1.53 (0.86–2.73)
GERD symptoms						
	Never	1.00 (Ref)	1.00 (Ref)	1.00 (Ref)	1.00 (Ref)	1.00 (Ref)
	Ever	5.24 (3.60–7.61)	7.50 (4.00–14.1)	4.27 (2.79–6.53)	3.85 (2.56–5.79)	13.0 (5.83–29.1)
GERD duration						
	Never	1.00 (Ref)	1.00 (Ref)	1.00 (Ref)	1.00 (Ref)	1.00 (Ref)
	<5 yrs	4.83 (1.08–21.7)	8.07 (1.37–47.6)	2.81 (0.52–15.2)	5.17 (1.01–26.4)	4.33 (0.68–27.5)
	5–9 yrs	4.09 (1.18–14.1)	5.97 (1.17–30.5)	3.53 (0.87–14.4)	2.84 (0.61–13.3)	8.73 (1.99–38.3)
	≥10 yrs	4.35 (3.09–6.12)	4.75 (2.93–7.69)	3.95 (2.68–5.82)	3.68 (2.51–5.40)	5.86 (3.52–9.75)
Frequency of GERD and age at onset						
	Never	1.00 (Ref)	1.00 (Ref)	1.00 (Ref)	1.00 (Ref)	1.00 (Ref)
	≥weekly age <30y	12.0 (5.95–24.3)	19.9 (7.96–49.7)	8.54 (3.85–18.9)	7.37 (3.36–16.2)	43.2 (14.9–125)
	≥weekly age 30-49y	7.77 (4.47–13.5)	12.0 (5.03–28.7)	6.01 (3.23–11.2)	5.71 (3.09–10.5)	19.6 (7.15–53.7)
	≥weekly age 50-79y	5.59 (3.15–9.90)	7.47 (3.12–17.9)	4.77 (2.49–9.13)	3.66 (1.90–7.05)	15.8 (5.89–42.5)
	<weekly age <30y	2.35 (0.64–8.67)	2.39 (0.25–22.8)	2.59 (0.62–10.7)	2.84 (0.77–10.4)	-
	<weekly age 30-49y	2.02 (0.56–8.67)	1.97 (0.21–18.1)	1.49 (0.30–7.51)	1.87 (0.46–7.58)	-
	<weekly age 50-79y	3.24 (1.01–10.4)	1.89 (0.21–17.2)	3.96 (1.16–13.5)	3.47 (1.02–11.8)	2.90 (0.29–29.2)
Smoking status						
	Never	1.00 (Ref)	1.00 (Ref)	1.00 (Ref)	1.00 (Ref)	1.00 (Ref)
	Ever	1.13 (0.79–1.62)	1.00 (0.60–1.65)	1.17 (0.77–1.79)	0.99 (0.66–1.49)	1.32 (0.77–2.26)
Alcohol status						
	Never	1.00 (Ref)	1.00 (Ref)	1.00 (Ref)	1.00 (Ref)	1.00 (Ref)
	Former	1.09 (0.57–2.07)	0.68 (0.30–1.56)	1.44 (0.64–3.22)	1.13 (0.53–2.39)	0.91 (0.38–2.21)
	Current	0.90 (0.48–1.69)	0.58 (0.26–1.26)	1.15 (0.52–2.53)	0.92 (0.45–1.92)	0.73 (0.31–1.72)
H pylori infection						
	No	1.00 (Ref)	1.00 (Ref)	1.00 (Ref)	1.00 (Ref)	1.00 (Ref)
	Yes	0.62 (0.44–0.88)	0.45 (0.26–0.79)	0.71 (0.48–1.05)	0.72 (0.49–1.07)	0.37 (0.20–0.69)
NSAID use						
	None	1.00 (Ref)	1.00 (Ref)	1.00 (Ref)	1.00 (Ref)	1.00 (Ref)
	< Daily	0.50 (0.22–1.15)	0.37 (0.09–1.49)	0.65 (0.26–1.61)	0.54 (0.22–1.37)	0.34 (0.07–1.69)
	Daily	0.91 (0.65–1.29)	0.86 (0.53–1.41)	0.91 (0.61–1.35)	0.78 (0.53–1.15)	1.15 (0.69–1.93)
PPI or H2RA use						
	No	1.00 (Ref)	1.00 (Ref)	1.00 (Ref)	1.00 (Ref)	1.00 (Ref)
	Yes	7.11 (5.02–10.1)	6.90 (4.20–11.3)	7.56 (5.03–11.3)	5.57 (3.79–8.20)	14.8 (8.06–27.3)
Active Gastritis						
	No	1.00 (Ref)	1.00 (Ref)	1.00 (Ref)	1.00 (Ref)	1.00 (Ref)
	Yes	0.78 (0.55–1.11)	0.72 (0.43–1.23)	0.82 (0.55–1.23)	0.95 (0.65–1.40)	0.45 (0.24–0.84)
Chronic Gastritis						
	No	1.00 (Ref)	1.00 (Ref)	1.00 (Ref)	1.00 (Ref)	1.00 (Ref)
	Yes	0.70 (0.51–0.95)	0.56 (0.35–0.88)	0.81 (0.56–1.15)	0.85 (0.60–1.21)	0.46 (0.28–0.74)

All BEs includes patients with specialized intestinal metaplasia on the study EGD, regardless of length and timing of diagnosis (new vs prevalent).

*Adjusted for age (<50, 50-<60, 60-<70, ≥70yrs), sex and race (white, non-white).

For comparisons with endoscopy controls, early onset and longer duration of GERD symptoms were statistically significantly associated with risk of long-segment BE but not short-segment BE. Likewise, *H*. *pylori* infection (OR, 0.54; 95% CI, 0.32–0.92) and chronic gastritis (OR, 0.65; 95% CI, 0.43–0.98) were associated with lower risk of long-segment BE but were not associated with short-segment BE (Table 2).

**Table 2:** Associations with risk of Barrett’s esophagus, compared with endoscopy controls.

		All BEs (n = 329)	Long-segment BE (n = 118)	Short-segment BE (n = 200)	Newly diagnosed BE (n = 208)	Prevalent BE (n = 109)
		AOR* (95% CI)	AOR* (95% CI)	AOR* (95% CI)	AOR* (95% CI)	AOR* (95% CI)
BMI						
	<25	1.00 (Ref)	1.00 (Ref)	1.00 (Ref)	1.00 (Ref)	1.00 (Ref)
	25–29.9	1.02 (0.71–1.47)	0.76 (0.43–1.35)	1.24 (0.79–1.94)	0.84 (0.54–1.29)	1.66 (0.87–3.16)
	≥30	1.16 (0.82–1.66)	1.10 (0.65–1.86)	1.27 (0.82–1.97)	1.07 (0.71–1.61)	1.55 (0.82–2.93)
WHR						
	Tertile 1	1.00 (Ref)	1.00 (Ref)	1.00 (Ref)	1.00 (Ref)	1.00 (Ref)
	Tertile 2	1.49 (1.07–2.06)	1.57 (0.94–2.62)	1.39 (0.94–2.06)	1.40 (0.95–2.07)	1.58 (0.92–2.70)
	Tertile 3	1.41 (1.03–1.93)	1.47 (0.89–2.41)	1.34 (0.91–1.95)	1.26 (0.87–1.84)	1.64 (0.98–2.74)
GERD symptoms						
	Never	1.00 (Ref)	1.00 (Ref)	1.00 (Ref)	1.00 (Ref)	1.00 (Ref)
	Ever	1.25 (0.89–1.77)	1.76 (0.96–3.22)	1.02 (0.68–1.52)	0.89 (0.61–1.31)	3.21 (1.46–7.04)
GERD duration						
	Never	1.00 (Ref)	1.00 (Ref)	1.00 (Ref)	1.00 (Ref)	1.00 (Ref)
	<5 yrs	1.71 (0.65–4.50)	2.63 (0.71–9.69)	1.27 (0.36–4.50)	1.87 (0.60–5.81)	1.71 (0.37–7.83)
	5–9 yrs	0.80 (0.34–1.84)	1.08 (0.31–3.71)	0.70 (0.24–2.04)	0.54 (0.16–1.82)	1.50 (0.50–4.53)
	≥10 yrs	1.42 (1.09–1.86)	1.63 (1.07–2.48)	1.29 (0.93–1.78)	1.20 (0.87–1.66)	1.98 (1.27–3.08)
Frequency of GERD and age at onset						
	Never	1.00 (Ref)	1.00 (Ref)	1.00 (Ref)	1.00 (Ref)	1.00 (Ref)
	≥weekly age <30y	1.53 (0.95–2.46)	2.94 (1.41–6.11)	1.01 (0.56–1.84)	0.91 (0.51–1.62)	5.33 (2.19–12.9)
	≥weekly age 30-49y	1.41 (0.91–2.18)	1.80 (0.86–3.78)	1.13 (0.68–1.89)	1.02 (0.62–1.69)	3.18 (1.30–7.75)
	≥weekly age 50-79y	1.09 (0.68–1.74)	1.36 (0.62–2.99)	0.93 (0.53–1.63)	0.68 (0.38–1.20)	3.24 (1.31–8.01)
	<weekly age <30y	0.96 (0.31–3.01)	0.99 (0.12–8.27)	1.00 (0.28–3.62)	1.18 (0.37–3.74)	-
	<weekly age 30-49y	0.76 (0.25–2.33)	0.86 (0.10–7.09)	0.53 (0.12–2.35)	0.70 (0.20–2.47)	-
	<weekly age 50-79y	1.12 (0.42–2.97)	0.71 (0.09–5.88)	1.32 (0.46–3.76)	1.15 (0.40–3.28)	1.20 (0.14–10.4)
Smoking status						
	Never	1.00 (Ref)	1.00 (Ref)	1.00 (Ref)	1.00 (Ref)	1.00 (Ref)
	Ever	1.15 (0.85–1.55)	1.07 (0.68–1.68)	1.15 (0.79–1.66)	1.00 (0.70–1.43)	1.35 (0.83–2.20)
Alcohol status						
	Never	1.00 (Ref)	1.00 (Ref)	1.00 (Ref)	1.00 (Ref)	1.00 (Ref)
	Former	1.08 (0.65–1.79)	0.62 (0.31–1.23)	1.55 (0.77–3.13)	1.17 (0.62–2.22)	0.86 (0.41–1.79)
	Current	1.25 (0.76–2.05)	0.76 (0.39–1.47)	1.74 (0.87–3.46)	1.35 (0.72–2.53)	0.98 (0.48–2.02)
H pylori infection						
	No	1.00 (Ref)	1.00 (Ref)	1.00 (Ref)	1.00 (Ref)	1.00 (Ref)
	Yes	0.72 (0.53–0.98)	0.54 (0.32–0.92)	0.81 (0.57–1.16)	0.85 (0.60–1.21)	0.43 (0.24–0.77)
NSAID use						
	None	1.00 (Ref)	1.00 (Ref)	1.00 (Ref)	1.00 (Ref)	1.00 (Ref)
	< Daily	0.91 (0.45–1.82)	0.66 (0.19–2.23)	1.12 (0.51–2.49)	1.01 (0.45–2.24)	0.55 (0.13–2.37)
	Daily	1.16 (0.87–1.55)	1.17 (0.76–1.82)	1.18 (0.83–1.67)	1.03 (0.74–1.45)	1.51 (0.95–2.40)
PPI or H2RA use						
	No	1.00 (Ref)	1.00 (Ref)	1.00 (Ref)	1.00 (Ref)	1.00 (Ref)
	Yes	1.33 (1.00–1.78)	1.24 (0.79–1.94)	1.42 (0.99–2.04)	1.03 (0.73–1.44)	2.76 (1.56–4.86)
Active Gastritis						
	No	1.00 (Ref)	1.00 (Ref)	1.00 (Ref)	1.00 (Ref)	1.00 (Ref)
	Yes	0.96 (0.71–1.30)	0.88 (0.54–1.44)	1.00 (0.70–1.44)	1.19 (0.84–1.69)	0.52 (0.29–0.94)
Chronic Gastritis						
	No	1.00 (Ref)	1.00 (Ref)	1.00 (Ref)	1.00 (Ref)	1.00 (Ref)
	Yes	0.78 (0.60–1.02)	0.65 (0.43–0.98)	0.88 (0.64–1.21)	0.96 (0.71–1.32)	0.51 (0.33–0.79)

All BEs includes patients with specialized intestinal metaplasia on the study EGD, regardless of length and timing of diagnosis (new vs prevalent).

*Adjusted for age (<50, 50-<60, 60-<70, ≥70yrs), sex and race (white, non-white).

Long-segment BE was more frequent among persons with prevalent BE than those with newly diagnosed BE (p<0.001). Among 109 cases of prevalent BE, 47 (43.1%) were short-segment BE and 62 (56.9%) were long-segment BE. Among the 208 cases of newly diagnosed BE, 152 (73.1%) were short-segment compared to 56 (26.9%) long-segment BE. Almost all the risk factors had higher risk estimates with long-segment BE vs. short-segment BE irrespective of new diagnosis or prevalent BE.

### Newly Diagnosed BE vs. Prevalent BE

Compared with primary care controls, patients with newly diagnosed BE were significantly more likely to have a history of early onset GERD symptoms (≥weekly GERD age <30 years: OR, 7.37; 95% CI, 3.36–16.2) or used PPIs or H2RAs (OR, 5.57; 95% CI, 3.79–8.20) (Table 1). There was a trend for lower risk of newly diagnosed BE with *H*. *pylori* infection (OR, 0.72; 95% CI, 0.49–1.07), although the association did not reach statistical significance. When we compared cases with prevalent BE to primary care controls, the associations with history of early onset GERD symptoms (≥weekly GERD age <30 years: OR, 43.2; 95% CI, 14.9–125) and use of PPIs/H2RAs (OR, 14.8; 95% CI, 8.06–27.3) were larger in magnitude than those for newly diagnosed BE. Likewise, the inverse association with *H*. *pylori* infection (OR, 0.37; 95% CI, 0.20–0.69) was stronger and statistically significant for risk of prevalent BE. Furthermore, active gastritis (OR, 0.45; 95% CI, 0.24–0.84) and chronic gastritis (OR, 0.46; 95% CI, 0.28–0.74) were associated with lower risk of prevalent BE but were not associated with newly diagnosed BE.

For comparisons with endoscopy controls, history of GERD symptoms, use of PPIs/H2RAs, high WHR, *H*. *pylori* infection and gastritis were associated with the risk of prevalent BE but not newly diagnosed BE (Table 2).

### Risk Factors for Endoscopy-only BE

We identified 85 patients with suspected endoscopic BE in the absence of SIM. Compared with primary care controls, patients with endoscopy-only BE were more likely to have a history of GERD symptoms (p = 0.003) and have used PPIs/H2RAs (p<0.001) (Table 3). In comparison with endoscopy controls, patients with endoscopy-only BE were less likely to have GERD symptoms (p = 0.02). There was some evidence for an association between higher WHR and endoscopy-only BE compared with primary care (OR, 1.64; 95% CI, 0.91–2.93) and endoscopy (OR, 1.65; 95% CI, 0.97–2.80) controls. Interestingly, endoscopy-only BE cases were less likely to be ever smokers than primary care (OR, 0.57; 95% CI, 0.34–0.97) and endoscopy (OR, 0.56; 95% CI, 0.35–0.91) controls. In comparisons with the definitive BE patient group (i.e., those with SIM present on histopathologic examination), patients with endoscopic BE only without SIM were less likely to have GERD symptoms (p = 0.004) and were less likely to have ever smoked (p = 0.01).

**Table 3:** Associations with risk of endoscopic-only Barrett’s esophagus.

		Endo-only BE *vs*. Primary care controls	Endo-only BE *vs*. Endoscopy controls
		AOR* (95% CI)	AOR* (95% CI)
BMI			
	<25	1.00 (Ref)	1.00 (Ref)
	25–29.9	0.62 (0.31–1.26)	0.76 (0.40–1.47)
	≥30	0.71 (0.37–1.37)	1.19 (0.66–2.15)
WHR			
	Tertile 1	1.00 (Ref)	1.00 (Ref)
	Tertile 2	0.73 (0.38–1.39)	1.06 (0.58–1.93)
	Tertile 3	1.64 (0.91–2.93)	1.65 (0.97–2.80)
GERD symptoms			
	Never	1.00 (Ref)	1.00 (Ref)
	Ever	2.25 (1.32–3.85)	0.54 (0.32–0.89)
GERD duration			
	Never	1.00 (Ref)	1.00 (Ref)
	<5 yrs	2.22 (0.21–23.2)	0.80 (0.10–6.17)
	5–9 yrs	5.14 (1.08–24.5)	1.11 (0.32–3.80)
	≥10 yrs	3.49 (2.07–5.91)	1.14 (0.70–1.84)
Frequency of GERD and age at onset			
	Never	1.00 (Ref)	1.00 (Ref)
	≥weekly age <30y	2.86 (0.88–9.26)	0.44 (0.17–1.12)
	≥weekly age 30-49y	3.81 (1.76–8.25)	0.64 (0.32–1.26)
	≥weekly age 50-79y	1.45 (0.50–4.16)	0.30 (0.11–0.81)
	<weekly age <30y	1.61 (0.19–13.8)	0.52 (0.06–4.07)
	<weekly age 30-49y	-	-
	<weekly age 50-79y	1.08 (0.12–9.58)	0.46 (0.06–3.63)
Smoking status			
	Never	1.00 (Ref)	1.00 (Ref)
	Ever	0.57 (0.34–0.97)	0.56 (0.35–0.91)
Alcohol status			
	Never	1.00 (Ref)	1.00 (Ref)
	Former	1.77 (0.52–6.08)	1.75 (0.60–5.11)
	Current	1.53 (0.45–5.15)	1.92 (0.67–5.50)
H pylori infection			
	No	1.00 (Ref)	1.00 (Ref)
	Yes	0.95 (0.56–1.62)	1.05 (0.64–1.72)
NSAID use			
	None	1.00 (Ref)	1.00 (Ref)
	< Daily	1.08 (0.38–3.11)	1.27 (0.48–3.38)
	Daily	0.68 (0.38–1.19)	0.81 (0.48–1.36)
PPI or H2RA use			
	No	1.00 (Ref)	1.00 (Ref)
	Yes	4.44 (2.61–7.54)	0.88 (0.53–1.44)
Active Gastritis			
	No	1.00 (Ref)	1.00 (Ref)
	Yes	0.76 (0.43–1.35)	0.95 (0.56–1.61)
Chronic Gastritis			
	No	1.00 (Ref)	1.00 (Ref)
	Yes	0.99 (0.61–1.62)	1.16 (0.74–1.82)

Endo-only BE are those with endoscopically suspected BE in the absence of specialized intestinal metaplasia.

*Adjusted for age (<50, 50-<60, 60-<70, ≥70yrs), sex and race (white, non-white).

## Discussion

In this large cross-sectional study with extensive characterization of BE patients we found that the strength and profiles of risk factors for BE varied according to the choice of comparison group (primary care vs. endoscopy controls) as well as with BE diagnosis timing (newly diagnosed vs. prevalent cases), endoscopic features of BE (long- vs. short-segment), and BE definition (SIM vs. endoscopic BE only without SIM). These findings are instructive for the understanding of BE risk factors, potential clinical risk stratification efforts, and epidemiological studies of BE [31–34]. GERD symptoms, especially those starting at young age, remain the single most important and consistent risk factor for BE; however, in some settings, new and exaggerated risk factors are likely to be identified.

Comparing BE cases to controls undergoing endoscopy for clinical indications is relevant for BE risk prediction in the setting of endoscopy. However, since most patients undergoing elective endoscopy have a history of GERD symptoms, few risk factors can predict (prior to endoscopic evaluation) which patients will have undiagnosed BE (i.e., a new BE case), and none of these factors are actionable in a clinical setting. We found that newly diagnosed BE patients were more likely to be white males, but only had slightly higher WHR and slightly lower rates of *H*. *pylori* infection compared to endoscopy controls. There were no differences in history of GERD symptoms, including early onset symptoms. These findings help explain why models that attempt to predict BE risk using information on a patient’s history of GERD symptoms, obesity and smoking, which are increasing common among patients presenting for endoscopy, have had poor discriminatory ability [31]. Conversely, being in care (i.e., prevalent or existing BE case) exaggerates the effect of some risk factors for BE. For example, we found even lower rates of *H*. *pylori* infection in prevalent BE cases relative to endoscopy controls and newly diagnosed BE cases; this may clarify the heterogeneity observed between studies examining the association with *H*. *pylori* infection where not all studies distinguished new from prevalent cases [10–12].

Comparing newly diagnosed BE cases with primary care (population-based) controls is a better setting to evaluate risk factors for BE screening. In our analysis, we found strong effects for white race, history of GERD symptoms, PPI/H2RA use and *H*. *pylori* infection. However, this is not a great setting for finding metabolic syndrome related risk factors (BMI, WHR), and our findings provide a rationale for why some, but not all, prior studies have observed associations of BMI and WHR with risk of BE [5–7]. Conversely, comparing primary care (population-based) controls with prevalent BE cases exaggerates (for example, by healthcare) the effect of risk factors that may not be important (*H*. *pylori* infection, gastritis, GERD symptoms, PPI/H2RA use). Additionally, comparison to primary care controls may be the worst setting to look for chemoprevention because the controls have equivalent risk factors for metabolic syndrome and increased medication use.

Longer segment length is one of the strongest predictors of neoplastic progression in patients with BE [21]. While data are limited, there is anecdotal evidence that risk profiles differ for long- vs. short-segment BE. Our findings are consistent with the hypothesis that risk factors for BE are stronger for long-segment BE than short-segment BE. While frequency and duration of GERD symptoms and use of PPIs/H2RAs were associated with both long-segment and short-segment BE in comparisons with primary care controls, the risk estimates for long-segment BE were of greater magnitude. Furthermore, *H*. *pylori* infection and chronic gastritis were only associated with long-segment BE. The differential magnitudes of these associations were also seen for comparisons with endoscopy controls. Therefore, these risk factors may be important for identifying higher risk patients for BE screening.

As expected, those recruited from primary care clinics who received endoscopy as part of this study represent mostly undiagnosed (new) cases of BE (25% of newly diagnosed BE cases came from primary care vs. 1% of prevalent BE cases from primary care), who would have been missed unless wide spread screening is implemented. Compared to primary care controls, those with newly diagnosed BE had more frequent and longer duration of GERD symptoms and more PPI/H2RA use. The magnitude of these risk factors were higher for prevalent than newly diagnosed BE cases. Those with prevalent BE were also less likely to have *H*. *pylori* infection, active or chronic gastritis, but these associations were not seen with newly diagnosed BE. This finding partly explains the lack of GERD symptoms in a considerable proportion of patients with esophageal adenocarcinoma and the relatively poor predictive value of GERD severity and duration in risk stratification for BE and esophageal adenocarcinoma [32,33].

Although endoscopy-only BE may not confer the same cancer risk as BE with SIM [35], a fraction of patients with endoscopy-only BE may have SIM that was missed due to sampling error, and some patients with CLE without SIM eventually get diagnosed with SIM [36]. It is therefore important to understand more about the pathophysiology of this potentially different entity of BE. We found those with endoscopic appearance of BE but without SIM were more likely than primary care controls to have GERD symptoms, longer duration of GERD symptoms, and PPI or H2RA use. However, in contrast to Balasubramanian et al [37] who found higher risk of columnar lined esophagus associated with longer duration of GERD symptoms among a cohort of GERD patients undergoing upper endoscopy, for comparisons with endoscopy controls, frequency and duration of GERD symptoms were not associated with increased risk of endoscopy only BE in our study.

There are several strengths of the current study. The large number of BE cases recruited into the parent study allowed, for the first time, the comparison of multiple BE case groups, and find significant differences in risk factors. BE cases were well characterized using extensive medical record review of histopathology and endoscopy reports to define length of BE and prevalent BE using strict and consistent criteria. Furthermore, surveys on demographics, GERD symptoms, and medication use were administered prior to EGD, thus reducing differential recall bias between cases and controls. This comprehensive survey allowed us to capture multiple dimensions of risk factor exposure, including at different age intervals. By including patients with concomitant dysplasia among both newly diagnosed and prevalent BE cases, we were able to capture more realistic associations for these risk factor and minimize incidence-prevalence bias. While our results may not be generalizable to women and non-veterans, because BE disproportionately affects men and whites, the veteran population is adequate to study risk factors for BE. Furthermore, our previously published results from this VA-based study have been generally consistent with results from BE studies conducted in the U.S., Ireland and Australia [5,17,18,25].

In summary, our results support the hypothesis that risk factors for BE are stronger (i.e., of greater magnitude) for long-segment than short-segment BE. The findings also support that epidemiological studies of BE should make a distinction between new and prevalent cases, long and short BE, endoscopic and histologically definitive BE as well as type of non-BE controls.

## Supporting Information

S1 Table Characteristics of Barrett’s esophagus cases and primary care controls. (DOCX)

S2 Table Characteristics of Barrett’s esophagus cases and endoscopy controls. (DOCX)
