# Supplementary material for: Risk Profiles for Barrett’s Esophagus Differ between New and Prevalent, and Long- and Short-Segment Cases
Source: PLoS One. 2016 Dec 30;11(12):e0169250. doi: 10.1371/journal.pone.0169250 (PMC5201279; doi:10.1371/journal.pone.0169250)
Supplement: S2 Table — (DOCX) [file pone.0169250.s002.docx]

**S2 Table. Characteristics of Barrett’s esophagus cases and endoscopy controls.**

|  |  | **Endoscopy controls,**  **N=1353** | **All BEs,**  **N=329** | ***P*** | **Long-segment BE, N=118** | ***p*** | **Short-segment BE, N=200** | ***p*** | **Newly diagnosed BE, N=208** | ***p*** | **Prevalent BE, N=109** | ***p*** | **Endo-only BE, N=85** | ***P*** |
| --- | --- | --- | --- | --- | --- | --- | --- | --- | --- | --- | --- | --- | --- | --- |
| Age, years, mean (SD) |  | 59.4 (8.6) | 61.6 (7.4) | <0.001 | 61.5 (7.1) | 0.01 | 61.7 (7.6) | 0.001 | 61.7 (6.6) | <0.001 | 61.5 (8.8) | 0.02 | 59.5 (7.8) | 0.98 |
| Male, n (%) |  | 1201 (88.8) | 321 (97.6) | <0.001 | 115 (97.5) | 0.003 | 195 (97.5) | <0.001 | 204 (98.1) | <0.001 | 105 (96.3) | 0.01 | 78 (91.8) | 0.39 |
| Race, n (%) |  |  |  | <0.001 |  | <0.001 |  | <0.001 |  | <0.001 |  | <0.001 |  | 0.08 |
|  | White | 857 (63.3) | 289 (87.8) |  | 112 (94.9) |  | 168 (84.0) |  | 177 (85.1) |  | 102 (93.6) |  | 64 (75.3) |  |
|  | Black | 452 (33.4) | 35 (10.6) |  | 5 (4.2) |  | 29 (14.5) |  | 30 (14.4) |  | 4 (3.7) |  | 19 (22.4) |  |
|  | Other | 44 (3.3) | 5 (1.5) |  | 1 (0.9) |  | 3 (1.5) |  | 1 (0.5) |  | 3 (2.7) |  | 2 (2.3) |  |
| BMI, n (%) |  |  |  | 0.35 |  | 0.27 |  | 0.37 |  | 0.33 |  | 0.13 |  | 0.23 |
|  | <25 | 266 (19.7) | 56 (17.0) |  | 22 (18.6) |  | 31 (15.5) |  | 40 (19.2) |  | 13 (11.9) |  | 16 (19.0) |  |
|  | 25-29.9 | 498 (36.9) | 117 (35.6) |  | 36 (30.5) |  | 77 (38.5) |  | 67 (32.2) |  | 46 (42.2) |  | 24 (28.6) |  |
|  | ≥30 | 586 (43.4) | 156 (47.4) |  | 60 (50.9) |  | 92 (46.0) |  | 101 (48.6) |  | 50 (45.9) |  | 44 (52.4) |  |
|  | Missing | 3 | 0 |  | 0 |  | 0 |  | 0 |  | 0 |  | 1 |  |
| WHR, n (%) |  |  |  | <0.001 |  | 0.01 |  | 0.02 |  | 0.02 |  | 0.01 |  | 0.05 |
|  | Tertile 1 | 499 (38.3) | 84 (25.8) |  | 28 (23.9) |  | 55 (27.8) |  | 58 (28.2) |  | 25 (23.2) |  | 25 (29.4) |  |
|  | Tertile 2 | 372 (28.6) | 110 (33.7) |  | 40 (34.2) |  | 65 (32.8) |  | 69 (33.5) |  | 36 (33.3) |  | 21 (24.7) |  |
|  | Tertile 3 | 432 (33.1) | 132 (40.5) |  | 49 (41.9) |  | 78 (39.4) |  | 79 (38.3) |  | 47 (43.5) |  | 39 (45.9) |  |
|  | Missing | 50 | 3 |  | 1 |  | 2 |  | 2 |  | 1 |  | 0 |  |
| GERD symptoms, n(%) |  |  |  | 0.12 |  | 0.03 |  | 0.77 |  | 0.72 |  | 0.001 |  | 0.02 |
|  | Never | 251 (19.5) | 49 (15.6) |  | 13 (11.4) |  | 35 (18.6) |  | 40 (20.6) |  | 7 (6.5) |  | 23 (30.3) |  |
|  | Ever | 1034 (80.5) | 264 (84.4) |  | 101 (88.6) |  | 153 (81.4) |  | 154 (79.4) |  | 100 (93.5) |  | 53 (69.7) |  |
|  | Missing | 68 | 16 |  | 4 |  | 12 |  | 14 |  | 2 |  | 9 |  |
| Smoking status, n (%) |  |  |  | 0.05 |  | 0.37 |  | 0.10 |  | 0.33 |  | 0.08 |  | 0.03 |
|  | Never | 375 (29.2) | 74 (23.6) |  | 29 (25.2) |  | 44 (23.4) |  | 50 (25.8) |  | 23 (21.3) |  | 31 (40.8) |  |
|  | Ever | 910 (70.8) | 240 (76.4) |  | 86 (74.8) |  | 144 (76.6) |  | 144 (74.2) |  | 85 (78.7) |  | 45 (59.2) |  |
|  | Missing | 68 | 15 |  | 3 |  | 12 |  | 14 |  | 1 |  | 9 |  |
| Alcohol status, n(%) |  |  |  | 0.35 |  | 0.61 |  | 0.14 |  | 0.36 |  | 0.88 |  | 0.41 |
|  | Never | 124 (9.7) | 23 (7.4) |  | 13 (11.4) |  | 10 (5.3) |  | 13 (6.7) |  | 10 (9.3) |  | 4 (5.3) |  |
|  | Former | 505 (39.6) | 120 (38.5) |  | 40 (35.1) |  | 75 (40.1) |  | 75 (38.9) |  | 40 (37.4) |  | 30 (39.5) |  |
|  | Current | 648 (50.7) | 169 (54.2) |  | 61 (53.5) |  | 102 (54.5) |  | 105 (54.4) |  | 57 (53.3) |  | 42 (55.3) |  |
|  | Missing | 76 | 17 |  | 4 |  | 13 |  | 15 |  | 2 |  | 9 |  |
| H pylori infection, yes, n (%) |  | 418 (30.9) | 68 (20.7) | <0.001 | 18 (15.3) | <0.001 | 47 (23.5) | 0.03 | 50 (24.0) | 0.04 | 14 (12.8) | <0.001 | 25 (29.4) | 0.77 |
| NSAID use, n (%) |  |  |  | 0.10 |  | 0.25 |  | 0.23 |  | 0.51 |  | 0.04 |  | 0.69 |
|  | None | 562 (49.0) | 119 (43.3) |  | 43 (43.4) |  | 71 (42.5) |  | 79 (45.1) |  | 35 (38.9) |  | 36 (51.4) |  |
|  | Less than Daily | 61 (5.3) | 11 (4.0) |  | 3 (3.0) |  | 8 (4.8) |  | 8 (4.6) |  | 2 (2.2) |  | 5 (7.1) |  |
|  | Daily | 523 (45.6) | 145 (52.7) |  | 53 (53.5) |  | 88 (52.7) |  | 88 (50.3) |  | 53 (58.9) |  | 29 (41.4) |  |
|  | Missing | 207 | 54 |  | 19 |  | 33 |  | 33 |  | 19 |  | 15 |  |
| PPI or H2RA use, n (%) |  |  |  | 0.02 |  | 0.15 |  | 0.03 |  | 0.63 |  | <0.001 |  | 0.80 |
|  | No | 414 (32.8) | 80 (25.8) |  | 30 (26.3) |  | 46 (24.7) |  | 60 (31.1) |  | 15 (14.2) |  | 26 (34.2) |  |
|  | Yes | 847 (67.2) | 230 (74.2) |  | 84 (73.7) |  | 140 (75.3) |  | 133 (68.9) |  | 91 (85.8) |  | 50 (65.8) |  |
|  | Missing | 92 | 19 |  | 4 |  | 14 |  | 15 |  | 3 |  | 9 |  |
| Active gastritis, n (%) |  |  |  | 0.12 |  | 0.09 |  | 0.40 |  | 0.97 |  | 0.002 |  | 0.53 |
|  | No | 964 (73.0) | 249 (77.3) |  | 93 (80.2) |  | 148 (75.9) |  | 148 (72.9) |  | 93 (86.9) |  | 64 (76.2) |  |
|  | Yes | 356 (27.0) | 73 (22.7) |  | 23 (19.8) |  | 47 (24.1) |  | 55 (27.1) |  | 14 (13.1) |  | 20 (23.8) |  |
|  | Missing | 33 | 7 |  | 2 |  | 5 |  | 5 |  | 2 |  | 1 |  |
| Chronic gastritis, n (%) |  |  |  | <0.001 |  | <0.001 |  | 0.05 |  | 0.12 |  | <0.001 |  | 0.91 |
|  | No | 648 (48.9) | 194 (60.3) |  | 77 (66.4) |  | 110 (56.4) |  | 111 (54.7) |  | 76 (71.0) |  | 41 (48.2) |  |
|  | Yes | 678 (51.1) | 128 (39.8) |  | 39 (33.6) |  | 85 (43.6) |  | 92 (45.3) |  | 31 (29.0) |  | 44 (51.8) |  |
|  | Missing | 27 | 7 |  | 2 |  | 5 |  | 5 |  | 2 |  | 0 |  |

Missing values were excluded from comparison tests.

All BEs includes all patients with specialized intestinal metaplasia (SIM) on the study EGD, regardless of length and timing of diagnosis (new vs prevalent).

Endo-only BE are patients with endoscopically suspected BE in the absence of SIM and were included as a separate case group (not included in “All BEs”).
